# Cloning, purification, and functional characterization of Carocin S2, a ribonuclease bacteriocin produced by *Pectobacterium carotovorum*

**DOI:** 10.1186/1471-2180-11-99

**Published:** 2011-05-12

**Authors:** Yung-Chieh Chan, Jian-Li Wu, Huang-Pin Wu, Kuo-Ching Tzeng, Duen-Yau Chuang

**Affiliations:** 1 Department of Chemistry, National Chung-Hsing University, 250, Kuokuang Rd., Taichung, 402, Taiwan; 2 Division of Pulmonary Medicine, Department of Internal Medicine, Chang Gung Memorial Hospital, Keelung, 204, Taiwan; 3 Department of plant pathology, National Chung-Hsing University, 250, Kuokuang Rd., Taichung, 402, Taiwan

## Abstract

**Background:**

Most isolates of *Pectobacterium carotovorum subsp. carotovorum *(Pcc) produce bacteriocins. In this study, we have determined that Pcc strain F-rif-18 has a chromosomal gene encoding the low-molecular-weight bacteriocin, Carocin S2, and that this bacteriocin inhibits the growth of a closely related strain. Carocin S2 is inducible by ultraviolet radiation but not by mutagenic agents such as mitomycin C.

**Results:**

A carocin S2-defective mutant, TF1-2, was obtained by Tn5 insertional mutagenesis using F-rif-18. A 5706-bp DNA fragment was detected by Southern blotting, selected from a genomic DNA library, and cloned to the vector, pMS2KI. Two adjacent complete open reading frames within pMS2KI were sequenced, characterized, and identified as caroS2K and caroS2I, which respectively encode the killing protein and immunity protein. Notably, carocin S2 could be expressed not only in the mutant TF1-2 but also in *Escherichia coli *DH5α after entry of the plasmid pMS2KI. Furthermore, the C-terminal domain of CaroS2K was homologous to the nuclease domains of colicin D and klebicin D. Moreover, SDS-PAGE analysis showed that the relative mass of CaroS2K was 85 kDa and that of CaroS2I was 10 kDa.

**Conclusion:**

This study shown that another nuclease type of bacteriocin was found in *Pectobacterium carotovorum*. This new type of bacteriocin, Carocin S2, has the ribonuclease activity of CaroS2K and the immunity protein activity of CaroS2I.

## Background

The phytopathogenic enterobacterium, *Pectobacterium carotovorum *subsp. *carotovorum*, is a phytoparasitic, Gram-negative, facultative anaerobic bacterium [1]. Pcc produces many extracellular pectic enzymes (pectate lyase, pectin lyase, exopolygalacturnoate lyase) and hydrolytic enzymes causing soft-rot disease, tissue maceration, and cell wall collapse [2,3]. The only current strategy against soft-rot disease involves chemical agents that unavoidably contaminate the environment [4]. Kikumoto *et al*. have demonstrated that mixed bacteriocin-producing avirulent strains of Pcc show high efficacy against soft-rot disease of Chinese cabbage [5].

Bacteriocins are bactericidal, extracellular toxins, produced by both Gram-positive and Gram-negative bacteria [6,7]. These proteinaceous molecules kill closely related bacteria. The susceptible cell is recognized by specific target receptors on the membrane, and the producer cell evades lethality by expressing a cognate immune protein. The colicin family produced by *Escherichia coli *is divided into DNase (colicins E2, E7, E8 and E9), RNase (colicins E3, E4 and E6), tRNase (colicins D and E5), and pore-forming colicins (colicins A, E1, Ia and Ib) [8]. Bacteriocins (especially nuclease bacteriocins) have a high amino acid sequence homology.

Natural bacteriocin molecules act via a number of mechanisms. For example, colicin E3 is a well-known ribonuclease that specifically cleaves 16S rRNA at the 3'-end of the coding sequence both *in vivo *and *in vitro*, which leads to the abolishment of protein synthesis resulting in death of the susceptible cell [9-12]. Previous reports indicate that colicin E3 consists of a killer protein with three domains (i.e., a translocation domain [T domain], receptor binding domain [R domain], and nuclease domain) and an immunity protein that retards antibiotic activity [13,14]. The R domain recognizes a specific receptor, BtuB on the cell membrane and the T domain interacts with the TolB protein in the cell periplasm of the sensitive cell to facilitate entry of the killer domain through the cell membrane. In addition to the attack mechanism, the immunity mechanism has been extensively elucidated. Notably the immunity protein and the killer protein interact initially at very high affinity because of charge attraction, and are separated at the cell surface through energy generated from the proton motif force [15-17].

In general, the C-terminal domain determines the type of bacteriocin. The C-terminal nuclease domains are not only interchangeable but also lack species specificity [18]. Strikingly, the tRNase type of bacteriocin may accelerate exhaustion of tRNA in the cytoplasmic pool and thereby impair protein synthesis *in vivo*. Ogawa *et al*. have demonstrated that particular tRNA molecules can be digested by colicin D as well as by colicin E5 [19,20]. It has been suggested that phage-associated klebicin D is a tRNase type of bacteriocin based on similarity to the nuclease-like domain of colicin D [21].

Nguyen *et al*. reported production of a high-molecular-weight bacteriocin (carotovoricin Er) and Chuang *et al*. reported production of a low-molecular-weight bacteriocin (LMWB; carocin) by *Pectobacterium*[22,23]. The former has a bulky antenna-like tail, inner core, and contractile cylindrical structure, and the carotovoricin-caused inhibition zone can be easily distinguished from that of carocin by its low diffusibility. Carocin S1 is a deoxyribonuclease type of LMWB (indicated by the letter S) and is secreted by Pcc strain 89-H-4. Additionally, export of Carocin S1 utilizes the type III secretion system in Pcc, which also controls the cell motility of the bacterium [24].

Pcc strain F-rif-18 is a spontaneous rifampin-resistant mutant of the wild-type 3F-3. Ultraviolet radiation can induce Pcc strain F-rif-18 to produce the LMWB Carocin S2. One of several sensitive cells, SP33, was selected as an indicator strain here. In the present study, the chromosomal bacteriocin gene, *carocin S2*, was introduced into an expression plasmid encoding two proteins, CaroS2K and CaroS2I. These proteins were purified and characterized and their primary activities of killing (CaroS2K) and immunity (CaroS2I) were investigated *in vivo *and *in vitro*.

## Results

### Isolation of Transposon Insertion Mutants

**Figure 1:**
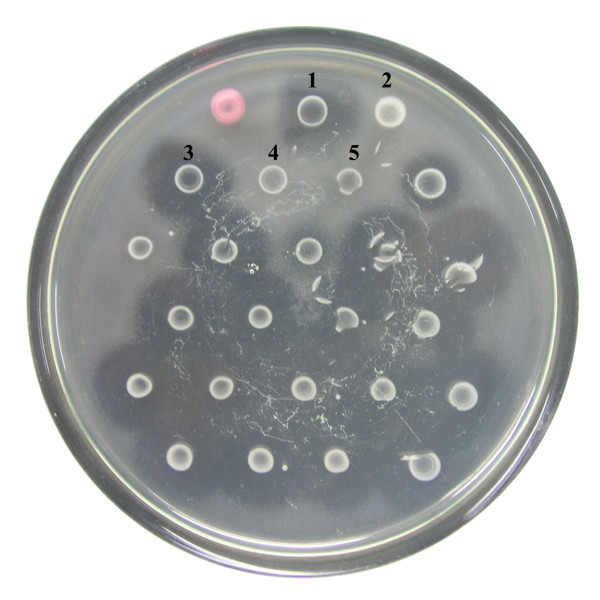
**Bacteriocin assays of Tn*5 *insertion mutants of Pcc strains**. Strain number: 1, 3F3 (wild type); 2, 1830 (*E. coli*); 3, F-rif-18 (parent); 4, TF1-1 and 5, TF1-2 (insertion mutant). Other unlabelled strains are Tn*5 *insertion mutants of F-rif-18 strain. The indicator is Pcc strain SP33.

Conjugation between F-rif-18 and *E. coli *1830 resulted in ~3,500 colonies after selection on Modified Drigalski's agar medium containing rifampin and kanamycin. In bacteriocin assay, the size of the inhibition zone around each isolate was compared with that of F-rif-18. Mutant colonies were identified by smaller inhibition zones. This evidence of mutation suggested that transposon Tn*5 *had been inserted into LMW bacteriocin-related genes. The strain TF1-2, a putative insertion mutant, would no longer produce LMW bacteriocin (Figure 1).

To ascertain whether Tn*5 *was actually introduced into the genomic DNA of putative isolates, the *nptII *gene of isolates was amplified using two primers P3 and P4 [23]. Southern blot technology showed that Tn*5 *had been inserted (Additional file 1, Figure S1).

### Identification of Tn5-inserted DNA Structures

**Figure 2:**
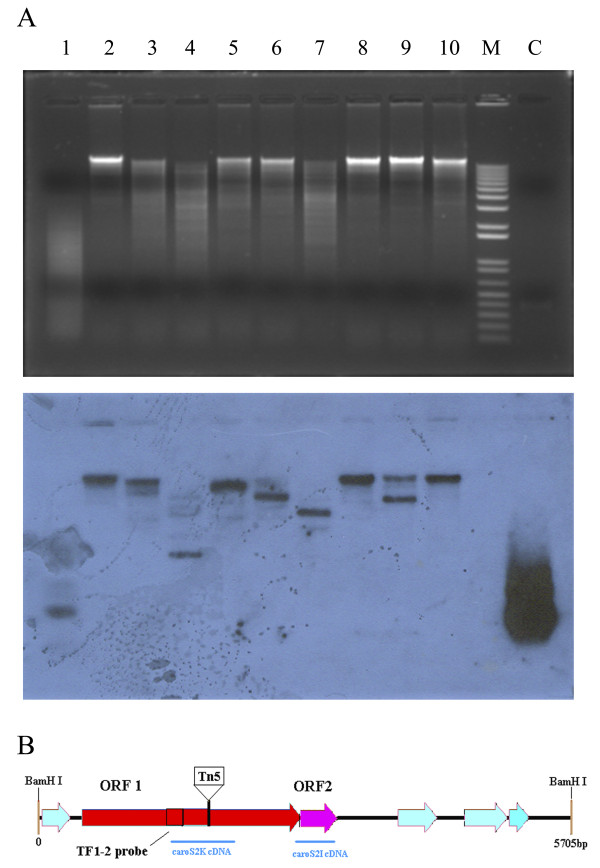
**DNA library screening and scheme of *carocin S2 *gene**. (A) The TF1-2 probe was used to screen DNA fragments from the genomic DNA library of F-rif-18. The DNA was digested with various restriction enzymes as follows: 1. Hpy188I; 2. *Hin*dIII; 3 HpaI; 4. *Eco*RV; 5. *Eco*RI; 6. *Cla*I; 7. *Bsa*AI; 8. *Bgl*II; 9. *Bam*HI; 10. *Ahd*I; M. DNA leader marker; C. The TF1-2 probe DNA. The arrowhead indicates the 5.7-kb carocin S2 fragment. (B) Shown is the 5.7-kb segment of DNA containing the *carocin S2*. The location of TF1-2 probe and part amplicon of cDNA of *caroS2K *and *caroS2I *were shown.

To identify Tn*5*-interrupted genes, genomic DNA from TF1-2 was amplified with TAIL-PCR using an array of specific primers (Additional file 1, Figure S8). A 2621-bp DNA fragment, including two open reading frames (ORFs), was identified as the sequence containing the bacteriocin structural gene. This gene was designated the *carocin S2 *gene. To characterize the *carocin S2 *gene, the TF1-2 probe was designed to hybridize in Southern blots with a *Bam *HI-digested DNA fragment from the genomic library of F-rif-18 (Figure 2A). A 5706-bp *Bam *HI-digested DNA fragment (Figure 2B), harboring two complete ORFs of *carocin S2*, was cloned into the plasmid pMCL210 (Additional file 1, Figure S2). The carocin-producing plasmid was designated as pMS2KI. The amplicon, comprising the predicted ORF2 of *caroS2I*, was subcloned into the pGEM-T easy vector, resulting in the plasmid pGS2I (Additional file 1, Figure S5).

### Transcriptional analysis and in vivo expression of carocin S2 gene

**Figure 3:**
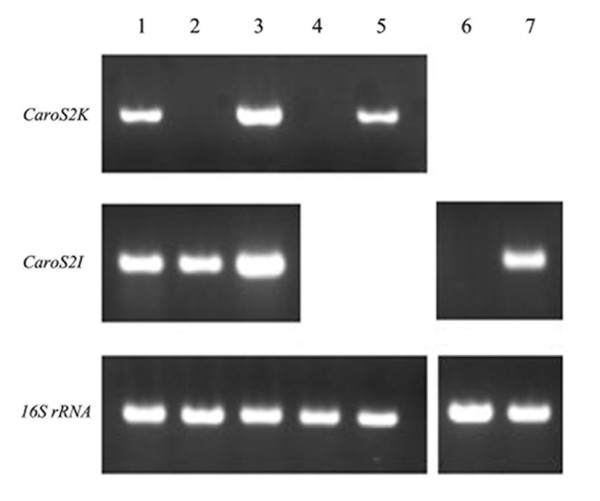
**Reverse Transcription PCR of RNA**. Shown are cDNA from the following strains: Lanes 1, F-rif-18; 2, TF1-2; 3, TF1-2/pMS2KI, 4, DH5α; 5, DH5α/pMS2KI.; 6, SP33; 7, SP33/pGS2I. The amplicons of *caroS2K *and *caroS2I *are 925 bp and 259 bp, respectively. The corresponding amplicons of 16S rRNA from the examined strains (lower panel). All samples were loaded equally.

To determine whether the *carocin S2 *gene is transcribed in a series of recombinant strains, reverse transcription-PCR was used to estimate RNA level. Two sets of intergenic primers were designed to amplify parts of transcripts from *caroS2K *or *caroS2I*, respectively (Figure 2B). Amplification of parts of 16S ribosomal RNA transcripts indicated that RNA in these bacterial cells is expressed at normal levels (Figure 3).

The presence of the 925-bp amplicon revealed that *caroS2K *was being transcribed in the cell (panel *caroS2K *in Figure 3). The TF1-2 strain, which is a Tn*5 *insertional mutant, could not transcribe *caroS2K *(lane 2), but the ability of TF1-2 to transcribe *caroS2K *was restored by introduction of pMS2KI (lane 3). It was apparent that the amount of *caroS2K *expression was dependent on the number of copies of plasmid pMS2KI (compare lane 1 to lane 3). Additionally, carocin S2 can be expressed in *E. coli *strain DH5α by introduction of pMS2KI (lane 4 and lane 5).

**Figure 4:**
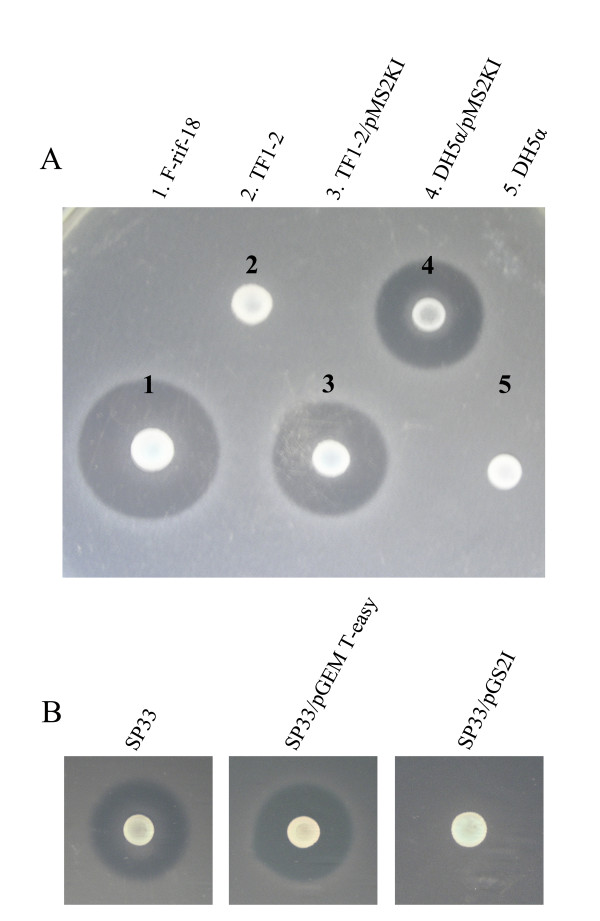
**Recovery and immunity activity of carocin S2**. (A) Antibacterial activity of carocin S2 from different strains. The indicator was Pcc strain SP33. Strain number: 1, F-rif-18; 2, TF1-2; 3, TF1-2/pMS2KI; 4, DH5α/pMS2KI; 5, DH5α. (B) Assay for *caroS2I*. The colony and inoculated strains were F-rif-18. The indicator strains were: 1, SP33; 2, SP33/pGEM-T easy; 3, SP33/pGS2I.

The presence of a 259-bp amplicon showed that *caroS2I *was transcribed constitutively (panel *caroS2I *in Figure 3). The *caroS2I *gene was transcribed unexpectedly in mutant strain TF1-2 even though the plasmid pMS2KI was introduced (lane 3). This demonstrated that *caroS2I *is expressed constitutively regardless of whether the gene *caros2K *is transcribed. Possibly an individual promoter for *caroS2I *gene is located behind the Tn*5 *insertion site in the *caroS2K *gene. CaroS2I transcripts were detected in strain SP33 with plasmid pGS2I (lanes 6 and 7). Although both the SP33 strains (with or without pGEM T-easy) were susceptible to Carocin S2, SP33/pGS2I appeared to grow in the presence of CaroS2K (Figure 4B).

To prove that pMS2KI contained the gene for Carocin S2, pMS2KI was introduced into TF1-2 and *E. coli *DH5α. Both TF1-2/pMS2KI and DH5α/pMS2KI had ability to express the activity of Carocin S2 (Figure 4A). The size of inhibition zone around strain TF1-2/pMS2KI was equal to that around DH5α/pMS2KI but still smaller than that around the wild-type strain F-rif-18. On the other hand, the quantity of transcripts expressed *in vivo *and *in vitro*did not usually correspond.

### Deduction of the amino acid sequence of Carocin S2

The *carocin S2 *gene consists of two ORFs (Additional file 1, Figure S7): one containing the 2352-bp *caroS2K *gene and the other containing the 273-bp *caroS2I *gene. The stop codon (TGA) of *caroS2K *overlaps the first start codon of *caroS2I *by 4-bp (ATGA). The amino sequences were deduced from the nucleotide sequence of the *carocin S2 *gene using DNASIS-Mac software (HITACHI, Japan) and compared to other analogous proteins using the BLAST and FASTA search tools.

**Figure 5:**
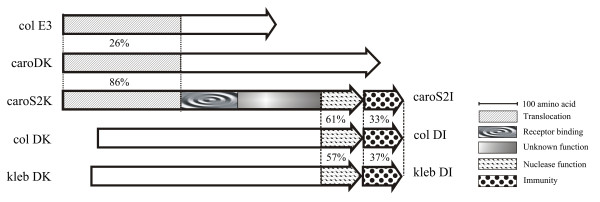
**Region similarity of the putative domains of carocin S2 with those of related bacteriocins**. The related ORFs are shown. Percentage values indicate the percent relatedness to the corresponding regions in carocin S2. The length of each domain is proportional to the number of amino acids. Homologous domains are shaded similarly. Domain I is homologous with the N-terminal T domain of colicin E3 [27]. Domain II resembles the receptor binding domains of other bacteriocins, but has no significant homology to other sequences in the database [8,30]. Domain III and ORF2 of carocin S2 are highly homologous to colicin D and klebicin D.

ORF1 was found to encode a 783-amino acid protein with a high degree of homology to Pcc21 carocin D, *Escherichia coli *colicin D and *Klebsiella oxytoca *klebicin D (Figure 5); ORF2 was found to encode a 90-amino acid protein that shows homology to the immunity proteins of colicin D and klebicin D (Figure 5). Thus, *caroS2K *produces an antibiotic with a deduced molecular mass of 85 kDa. CaroS2I (a 10-kDa protein of 90 amino acids) was shown to confer resistance to CaroS2K. It is particularly noteworthy that the homology between CaroS2K and Colicin D and Klebicin D is at the C-terminal end of these proteins where the catalytic center of a ribonuclease is located. According to the FASTA program, the amino acid segment between Asp677 and the C-terminus of CaroS2K shares almost 60% similarity with the minimal tRNase domain of colicin D and klebicin D (Figure 5). Since the colicin D and klebicin D are well-known tRNase family of bacteriocins, suggests that Carocin S2 might therefore be a ribonuclease.

### Purification and characterization of Carocin S2

**Figure 6:**
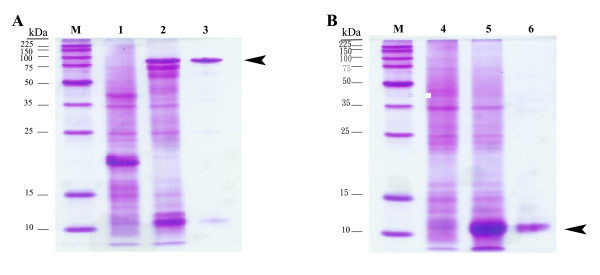
**SDS-PAGE analysis of purified protein**. Shown are the CaroS2K (A) and CaroS2I (B). Samples were subjected to electrophoresis in 10% polyacrylamide gels, which were stained with Coomassie blue. Lane M, molecular weight standards (kDa); lane 1, cell lysate of *E. coli *BL21/pET32a; lane 4, cell lysate of BL21/pET30b; lanes 2 and 5, IPTG-induced cell lysates of BL21/pES2kI and BL21/pES2I, respectively; lanes 3 and 6, purified protein obtained after elution. The arrowheads indicate the killing protein of carocin S2K (A) and the immunity protein of carocin S2I (B).

*E. coli *BL21 (DE3) recombinants, which were transformed with pES2KI or pES2I, were used to express CaroS2K protein or CaroS2I protein individually. Coomassie blue stained SDS-PAGE gels of purified Carocin S2 are shown in Figure 6. The band corresponding to CaroS2K was purified. The gel indicates a relative mass (M_r_) of about 85 kDa (Figure 6A), enrichment of the purified CaroS2K (arrowhead), and disappearance of other bands. Purification of CaroS2I by the same procedure resulted in a more intense band in the region of M_r _10 kDa (arrowhead; Figure 6B).

**Figure 7:**
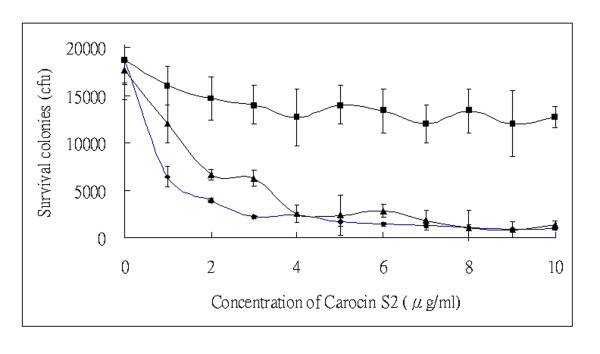
**Survival of SP33 cells treated with Carocin S2**. Aliquots of indicator SP33 cells were treated with increasing concentrations of CaroS2K (◆) and CaroS2K:CaroS2I in molar ratio of 1:1 (▲). The effect of trypsin on the CaroS2K was also assayed (■). The data are reported as means ± standard deviations.

The purified CaroS2K involved in the growth inhibition of the susceptible indicator strain SP33 was then characterized. The number of viable cells decreased with increasing concentration of CaroS2K (Figure 7). Almost all cells were dead at the initial concentration of 4 μg ml^-1^, indicating that about 90% of indicator strains are killed at this concentration. However, the activity of CaroS2K was inhibited by trypsin, but not inhibited by CaroS2I.

### Carocin S2 has ribonuclease activity

**Figure 8:**
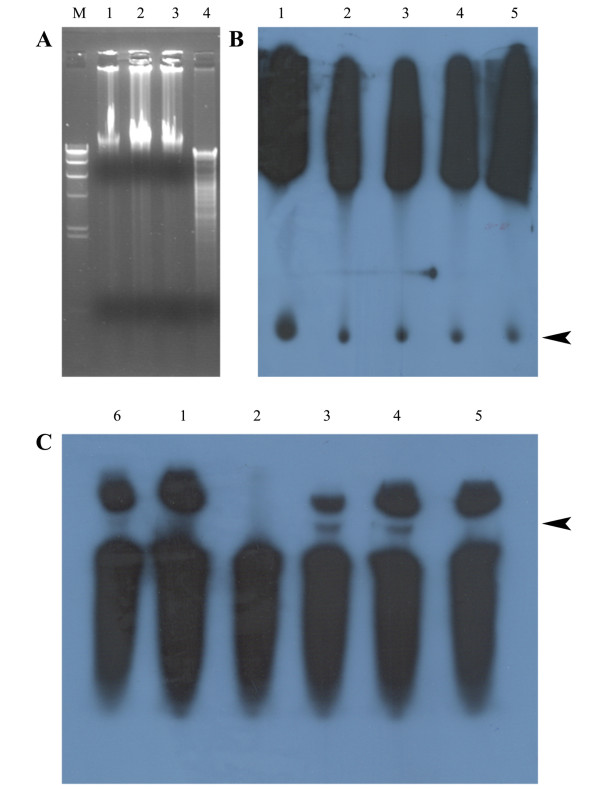
***In vitro *hydrolysis of DNA and RNA by Carocin S2**. (A) Analysis of the DNase activity of carocin S2. Lane M, the *Hin*dIII-digested λ DNA marker; lane 1, genomic DNA only; lanes 2 and 3, genomic DNA treated or untreated with carocin S2 in buffer, respectively; lane 4, equal quantity of *Eco*RI-digested genomic DNA. The 5'-labeled total RNA (B) and 3'-labeled total RNA (C) (1 μg of RNA per sample) were incubated without (lane 1) or with 1 μg (lane 2), 100 ng (lane 3), 10 ng (lane 4), or 1 ng (lane 5) of Carocin S2 and the result was assessed by autoradiography. The arrowhead indicates that the RNA segment digested from ribosome. Equal amounts of Carocin S2I and Carocin S2K mixed before RNA digestion (lane 6).

In order to confirm the role of carocin S2 as a ribonuclease type bacteriocin, we set up a RNA degradation assay. Northern blots of 5'-^32^P-labeled total RNA extract treated with increasing concentrations of CaroS2K (Figure 8B) showed a markedly lower intensity of labeled RNA fragments compared to untreated extracted RNA (Figure 8B, lane 1), suggesting that CaroS2K has ribonuclease activity.

Surprisingly the RNA segments were larger when the RNA was 3'-^32^P-labeled compared with 5'-^32^P-labeling (Figures 8B and 8C). As the concentrations of 23S RNA and 16S RNA decrease on the addition of increasing concentrations of CaroS2K, it is assumed that more ribosomal RNA is degraded leaving material that is ostensibly the ribosome. When excess concentrations of caroS2K (i.e 1 μg) are added then most of the ribosomal RNA is degraded leading to a destabilization and subsequent degradation of the ribosome (Figure 8C, lane 2). We hence consider that CaroS2K (in sufficient amount) would degrade the ribosome. CaroS2I inhibits the killing activity of CaroS2K because a mixture of equal quantities of CaroS2K and CaroS2I prevented digestion of RNA segments by CaroS2K (Figure 8C, lane 6).

Subsequently, treatment of the genomic DNA of the indicator strain SP33 with the purified CaroS2K protein had no effect on deoxyribonuclease activity, as compared to the pattern of *Eco*RI-digested genomic DNA (Figure 8A and Additional file 1, Figure S4).

### Nucleotide sequence accession number

The Genbank accession number of the sequence of the *carocin S2 *gene is HM475143.

## Discussion

In this study, a chromosome-borne gene encoding bacteriocin, carocin S2, in Pcc strain 3F3 was shown to possess ribonuclease activity. According to Bradley's classification, Carocin S2 is a low-molecular-weight bacteriocin [25]. Two genes, *caroS2K *and *caroS2I*, encode the 85-kDa and 10-kDa components, respectively, of Carocin S2. The substrate and gene structure of carocin S2 were unlike those of other bacteriocins from Pcc.

On the basis of sequence analysis, *carocin S2 *comprises these two overlapping ORFs, *caroS2K *and *caroS2I *(Additional file 1, Figure S7). A putative Shine-Dalgarno sequence 5'-AUGGA-3', which has also been seen in the DNA sequence of carocin S1, is located upstream (-9 bp to -13 bp) of the start codon AUG, suggesting that it could be a ribosome binding site for *caroS2K *[23]. Comparison of the upstream sequences of both *caroS2K *and *caroS2I *has shown that the two consensus sequences, 5'-TATAAAAA-3' (-34 bp to -41 bp) and 5'-GAAGT-3' (-61 bp to -65 bp), are both upstream from the start codon. Presumably, 5'-TATAAAAA-3' is the -10 promoter and 5'-GAAGT-3' is the -35 promoter for the carocin S2 gene, even though they differ from those of *E. coli*[26].

A putative -10 promoter is 33 bp upstream from the initiator ATG of the *caroS2K *gene, in which the SD sequence is embedded, while the -35 promoter is 19 bp upstream of the -10 promoter region. The putative promoter of the -35 box of *caroS2I *is located similarly near the -10 box, but the -10 box is just 24 bp upstream of the start codon where no SD sequence is apparent. Although those hypothesized promoters are located within the *caroS2K *structural gene, transcripts of *caroS2I *are routinely produced (Figure 3). This suggests that *caroS2I *RNA expression may be regulated posttranscriptionally, in spite of close neighboring genes downstream of the gene *caroS2K*; that is, core promoter elements may influence the expression of *caroS2I *gene.

In the present study, we attempted to separate CaroS2K from CaroS2I attached to (His)_6_-tag using a Nickel column (pEH2KI; Additional file 1, Figure S5), but a small amount of CaroS2I (Mr ~10 kDa) was observed in SDS-PAGE gels (Figure 6, bottom in lane 3), which had little influence on the activity of CaroS2K as the purified protein still had transient killing activity. Additionally, the activity of the Carocin S2 complex at 4℃ was long-lasting indicating good stability.

The C-terminal amino acid sequence of Carocin S2 had higher homology to those of colicin D and klebicin D, which are produced by *E. coli *and *Klebsiella oxytoca*, respectively, than to the amino acid sequence of carocin S1 from the same species (Additional file 1, Figure S6B).

The amino acid sequence of CaroS2K has three putative domains. Domain I (the N-terminal 314-residue sequence ending in Pro314) is regarded as the translocation domain and is homologous to the translocation domains of carocin D and colicin E3 (Figure 5). It is assumed to direct the cytotoxic domain to the periplasmic space [27,28]. Additionally, the putative TonB box (a sequence recognition motif DTMTV) was found in the N-terminal domain of CarocinS2, which is thought to participate in bacteriocin translocation [8]. Thus, we suggested that Carocin S2 could be a TonB-dependent bacteriocin.

Domain III (extending from Asp677 to the carboxyl terminus) is the killer domain. Particularly noteworthy is the resemblance of the killer domain to the tRNase domain of colicin D and klebicin D (Figure 5), and thus we suggested that carocin S2 might have tRNase activity [29-31]. Domain II extends 141 residues from Ilu315 to Val455 and is hypothesized to be the binding site that recognizes specific receptors on cell membranes. Additionally, domain III has no significant homology to carocin D, suggesting that carocin S2 and carocin D have different functions [28].

Finally, we showed that total RNA (whether labeled with radioactive phosphate at the 5'- or at the 3'- end) is sensitive to Carocin S2. Carocin S2 degraded 5'-labeled total RNA but not 5'-labeled CaroS2K-free RNA (Figure 8B), and the amount of degradation was not dose-dependent (arrowhead). However, the appearance of segments of unknown origin paralleled partial degradation of 23S and 16S rRNA (Figure 8C). These results suggest that the site of excision (either conformational or sequential) is close to the 5'-terminus of rRNA. Notably, the decrease in the amount of rRNA depended on the amount of Carocin S2 protein present, with complete degradation occurring in the presence of excess Carocin S2. Ogawa *et al*. reported that RNase type of bacteriocins, colicin E3 and colicin E5, catalyze the hydrolysis of the shorter RNAs from 16S rRNA [19,32]. Moreover, colicin E5 was found to hydrolyze tRNA *in vitro*. Furthermore, it was previously reported that colicin E3 cleaved 16S rRNA completely, and even 30S rRNA [11,33]. In our study, carocin S2 acted as an RNase that hydrolyzes rRNA (both 23S and 16S) *in vitro*. In terms of enzymatic function, Carocin S2 may act as an endo- and exo-ribonuclease simultaneously. Moreover, CaroS2I significantly inhibited nuclease activity *in vitro *but not *in vivo *(Figures 7, Figure 8 andAdditional file 1, Figure S3). We speculated that immunity protein CaroS2I might not be able to cross the cell membrane, as previously described [14]. Although our *in vitro *experiment showed that carocin S2 was a ribonuclease, further investigation is needed to clarify its function in cells.

One of the other Tn*5 *insertional mutants, TF1-1, which disrupted the coding sequence of the *fliC *gene, was found to halt expression of Carocin S2 (Figure 1), indicating that Carocin S2 can also be secreted via the type III secretion system [24]. The role of carocin S2 as an RNase in the cytoplasm is to prevent protein synthesis by cleaving either 23S rRNA or 16S rRNA. The role of the immunity protein, CaroS2I, is usually to stop the damage caused by CaroS2K in the cytoplasm. More details of the actual mechanism of carocin S2 remain to be elucidated.

## Conclusion

As shown herein, the novel bacteriocin, Carocin S2, was characterized as a ribonuclease. It is the first bacteriocin with ribonuclease activity to be found in *Pectobacterium *strains. We suggested that Carocin S2 kills the indicator cell by exhausting its supply of some kinds of RNA, leading to inactivation of protein biosynthesis. It will be of interest to study the proteomics of Carocin S2 and its mechanism of action in the future.

## Methods

### Bacterial strains, media, and growth conditions

Bacterial strains and plasmids used in the study are listed in Table 1. Isolates of Pcc were grown at 28°C in Luria-Bertani (LB) medium or IFO-802 medium. The IFO-802 medium was supplemented with 1% polypeptin, 0.2% yeast extract, 0.1% MgSO_4 _(pH 7.0), and 1.5% agar. Isolates of Pcc were distinguished from *Escherichia coli *by their ability to grow on Modified Drigalski's agar medium [34]. Antibiotics (final concentration, 100 μg ml^-1 ^of media) were added when necessary.

**Table 1:** Bacteria and plasmids used in the study

Strain or plasmid	Description	Source
***Escherichia coli***		
1830	*pro¯ met¯ Kan^r ^Nm*^r^, containing transposon *Tn5 *on the suicidal plasmid pBJ4JI	[44]
DH5α	supE44ΔlacU169(Φ80lacZΔM15) hsdR17recA1 gyrA96thi-1relA1	[39]
BL21(DE3)	hsdS gal(λ*c*I*ts*857 *ind*1 Sam*7 nin5 *l*ac *UV5-T7 gene 1)	[45]
***Pectobacterium carotovorum *subsp. *carotovorum***		
3F-3	Pcc, wild-type	Laboratory stock
F-rif-18	3F3, Rif^r^, wild-type	This study
TF1-1	F-rif-18, *fliC*::Tn*5*, Rif^r^, Kan^r^	This study
TF 1-2	F-rif-18, *Carocin*S2::Tn*5*, Rif^r^, Kan^r^	This study
SP33	Pcc, wild-type	Laboratory stock
**Plasmid**		
pMCL210	p15A, Cml^r^, Low copy number	[46]
pGEM T-Easy	Amp^r^; lacZ cloning vector	Promega
pET32a	Amp^r^; expression vector with the N-terminal His-tag	Novagen
pET30b	Kan^r^; expression vector with the C-terminal His-tag	Novagen
pMS2KI	5.7-kb BamHI DNA fragment harboring *carocin *S2 gene from 3F3 genome, cloned into pMCL210	This study
pEN2K*	*caroS2K *subcloned into pET32a	This study
pES2KI	Derived from pEN2K; deleted series of Tag element in front of expressed *caroS2K*	This study
pEH2KI*	Derived from pES2KI; adding (His)_6_-Tag adjacent to *caroS2I*	This study
pGS2I	*caroS2I *and its putative promoter from pMS2KI, subcloned into pGEM T-easy	This study
pECS2I*	*caroS2I *subcloned into pET30b, but the expressed fusion CaroS2I has no activity	This study
pES2I	Derived form pECS2I, the (His)_6_-Tag element was deleted	This study

Kan^r^: Kanamycin; Cml^r^: Chloramphenicol; Rif^r^: Rifampicin; Amp^r^: Ampicillin.

*: See Additional file 1, Figure S5.

### Bacterial conjugation

Overnight cultures of Pcc (recipient) and *E. coli *(donor) were mixed and spread onto 0.22-μm membrane filters placed on LB agar media and incubated overnight at 28°C [23]. The progeny after conjugation were appropriately diluted and cultivated on Modified Drigalski's medium (with ampicillin and kanamycin [100 μg ml^-1^]) overnight at 28°C. All isolates were placed on IFO-802 medium and tested for bacteriocins. Bacteriocin was assayed using the double-layer method, and Pcc SP33 was used as indicator strain [35]. The cells were incubated for 12 hours to form colonies, exposed to ultraviolet irradiation, incubated again for 12 hours, treated with chloroform to kill the cells, and then covered with soft agar containing indicator cells. The bacteriocin production was indicated by a zone of inhibition of indicator-cell (SP33) growth around the colony.

### Genetic-engineering technique

The procedures of plasmid preparation, genomic DNA isolation, and DNA manipulation were performed as described by Sambrook et al. [36]. Oligonucleotide DNA primers were synthesized by MD Bio Inc. (Taipei, Taiwan). The PCR was amplified with Go-Taq DNA polymerase (Promega, USA). The thermal asymmetric interlaced PCR (TAIL-PCR) was performed as previously described [37].

Plasmids were introduced into Pcc strains using electroporation (1.25 kV/cm, 200 Ω, 25 μF) [38]. For heat-shock transformation, the competent cells of *E. coli *were prepared according to the method of Hanahan [39].

Exponentially growing cells (OD_595 _of about 6.0) were harvested for RNA preparation. Total RNA was isolated using Trizol reagent (Invitrogen, USA) according to the manufacturer's instructions. RNA was resuspended in diethylpyrocarbonate (DEPC)-treated water. The concentration of RNA was determined by OD_260 _absorption, and RNA was analyzed by electrophoresis on 1.5% formaldehyde-morpholinepropanesulfonic-agarose gel.

Reverse transcription-PCR (RT-PCR) was carried out with AMV Reverse Transcriptase (Promega Inc., Taiwan) according to manufacturer's instructions. RNA (1 μg) was subjected to RT-PCR containing CaroS2_re_1 used as a reverse primer in first-strand cDNA synthesis. The RT mixtures were diluted and used as templates in a PCR reaction with two primers CaroS2_re_1 and CaroS2_for_1 (Additional file 1, Table S1).

A 2621-bp *BamH*I-*Hin*dIII digested DNA fragment, including the *caroS2K *and *caroS2I *genes, was amplified from pMS2KI with primers of CarocinS2K_for2 and CarocinS2I_rev2 (Additional file 1, Table S1) and subcloned into pET32a to give the plasmid pEN2K (Additional file 1, Figure S5). The pES2KI was obtained by excision of the Tag element between the rbs (ribosome binding site) and start code (for CaroS2K) in pEN2K using the SLIM method as previously described [40,41]. The 5IHT32a2KI_forT, 5IHTGT2KI_forS, 5IHT32a3KI_revT, and 5IHT32a4KI_revS primers were used. A 273-bp fragment of the caroS2I gene was amplified by PCR and ligated into the *Nde*I and *Xho*I site of pET30b to form the plasmid pEC2I. Similarly, the plasmid pES2I was obtained by deleting the (His)_6_-tag of pEC2I (carried out as described above with primers of X21_forT, X21_forS, X21_revT and X21_revS). Subsequently, pES2KI and pES2I were introduced into *E. coli *BL21 (DE3) cells, respectively.

### Restriction DNA library screening and Southern blots

Southern blots were performed according to the DIG Application Manual (Roche, USA). A 543-bp DNA fragment (TF1-2 probe) was amplified with TF1-2P and TF1-2A2 primers (Additional file 1, Table S1), subcloned into pGEM-T Easy vector (Promega Inc., USA), and labeled using a Random Primed DNA Labeling Kit (Roche Diagnostics, USA).

The genomic DNA of the wild-type strain F-rif-18 was digested with various restriction endonucleases, with sites located outside the putative open reading frame. Samples were electrophoresed and analyzed with Southern blotting. After detection using the TF1-2 probe, the DNA from positive gel slices was purified and cloned into pMCL210 to give the carocin-producing plasmid pMS2KI. The pMS2KI construct was isolated and detected as above with the TF1-2 probe.

### Protein purification

The transformant cells of BL21, harboring pES2KI or pES2I, were grown in 500 ml to an OD_595 _of 0.4. The cells were induced with isopropyl-β-D-thiogalactopyranoside (IPTG; final concentration, 0.1 mM; at 25°C for 12 h). Subsequently, the cells were pelleted and the pellets were sonicated (10 cycles of 9 s with 9-s intervals). BL21/pES2KI pellets were subjected to ammonium sulfate precipitation (30-40%), resuspended in buffer A (30 mM NaCl and 20 mM Tris-Cl, pH 8.0), and applied to a Fractogel column (Merck, USA). The fraction was eluted by a NaCl gradient (30 mM-1.4 M). After purification through a P-100 size-exclusion column (BioRad, USA), the CaroS2K fractions were pooled and concentrated using an Amicon centriprep-50 column (Millipore, USA) and dissolved in buffer A. BL21/pES2I pellets were precipitated by ammonium sulfate (70-100%) and resuspended in buffer A. CaroS2I purification involved a similar chromatographic procedure using the Amicon centriprep-3 column (Millipore, USA). The concentration of protein was determined by the Bradford assay (Amresco, USA).

### In vitro determination of Carocin S2 activity

Total RNA was treated with calf intestinal alkaline phosphatase (Promega, USA) at 55°C for 30 min as recommended by the manufacturer. The reaction was arrested by adding 5 mM nitrilotriacetic acid, and RNA was extracted with equal volumes of phenol/chloroform. An aliquot of phosphatase-treated RNA was 5'-^32^P-labeled at 37°C for 30 min by incubation with a mixture of [γ-^32^P]ATP, T4 polynucleotide kinase (Promega Inc, USA), and reaction buffer in nuclease-free water [42]. [5'-^32^P]Cytidine 3',5'-bisphosphate (pCp) and T4 RNA ligase (Promega, USA) were used for 3'-labeling of RNA [43]. Subsequently, the mixture was purified by MicroSpin G-25 columns (GE Healthcare, USA). The purified labeled RNA was divided into aliquots and incubated without or with Carocin S2 at 28°C for 60 min, respectively. To measure its activity, CaroS2I was pre-mixed with an equal amount of CaroS2K. The mixtures were subjected to electrophoresis on a 9% polyacrylamide gel (19:1) containing 7M urea, 50 mM Tris, 50 mM boric acid, and 1 mM EDTA, pH 8.3. All samples were electrophoresed at 15℃ by PROTEIN II xi (BioRad, USA).

To confirm DNase activity, 1 μg of genomic DNA from SP33 in solution containing buffer A was incubated with or without Carocin S2 at 28°C for 90 min. An equal quantity of genomic DNA was digested with *Eco*RI at 28°C for 90 min. Samples were then subjected to electrophoresis on 1% agarose gel.

### Antibiotic activity of Carocin S2

Overnight cultures of SP33 were diluted (1:100) with LB medium and grown at 28°C to a density of approximately 10^5 ^CFU ml^-1^. The activity of increasing concentrations of Carocin S2 on cells in suspension incubated at 28°C for 60 min was assessed. CaroS2I was pre-mixed with an equal molar ratio of CaroS2K. All reaction mixtures were spread onto LB agar plates and incubated at 28°C for 16 h. The experiment was performed three times. Colonies growing on a series of plates were respectively counted.

### Computer analysis of sequence data

Sequencing of the DNA fragments was carried out using an ABI automated DNA sequencer 373S. The nucleotide sequence data were compiled by DNASIS-Mac software (Hitachi, Japan). Amino acid sequences were compared using international BLAST and FASTA servers. Also, the putative domains of Carocin S2 were predicted using the PSI/PHI-BLAST.

## Authors' contributions

YC participated in the discovery and characterization of Carocin S2, and he wrote this manuscript. JL participated in protein purification. HP participated in manuscript preparation. KC supported the Pcc strain SP33 and for insightful discussion and guidance. DY conceived of the study, participated in its design, and corrected the manuscript. All authors read and approved the final version of the manuscript.

## Supplementary Material

Additional file 1 **Figure S1. Analysis of Tn5 insertional mutants by southern blotting**. Lane M, the HindIII-digested λ DNA marker; the genomic DNA of strains were loading as follows: lane 1, TF1-2; lane 2, F-rif-18; lane 3, 3F3; lane 4, TF1-1. Lane 5, the construct pGnptII that contain the detect probe DNA nptII. The result shows that TF1-2 and TF1-1 was a Tn*5 *insertional mutant. **Figure S2. The construct pMS2KI was cloned from genomic DNA library and screening by southern blotting with TF1-2 probe**. By southern blotting, it showed that the *carocin S2 *has been cloned to form pMS2KI. **Figure S3. The total RNA of SP33 were digested with Carocin S2 and electrophoresis **as follows: lane 1, RNA (1 μg); lane 2, RNA and CaroS2K (20 μg); lane 3, RNA and CaroS2I (4 μg); lanes 4 to 6 are RNA (1 μg) and CaroS2K (20 μg) with gradient concentration of CaroS2I, which were added with 4 μg (lane 4); 20 μg (lane 5); 100 μg (lane 6). All reactions were performed at 28℃ for 3 hours. **Figure S4. Metal effect of In vitro hydrolysis of DNA by Carocin S2**. Lane M, the *Hind*III-digested λ DNA marker; lane 1, the genomic DNA of SP33 only; lane 2, the *Eco*RI-digested genomic DNA; the genomic DNA was incubated with Carocin S2 (lane 3 to 5), or not. Magnesium acetate, nickel acetate and zinc acetate was added in buffer A (pH = 7), respectively. The reactions were performed at performed at 28℃ for 1 hour. **Figure S5. Schematic representation of the cloning strategy used in this study**. (1) A 543-bp amplicon was cloned into the vector pTF1 to form the pTF1-2-probe. (2) The TF1-2 probe was prepared. (3) The multi-enzyme-digested DNA fragments were obtained from F-rif-18 genomic DNA, and they were detected on southern blots. (4) Positive cDNA was cloned into the carocin-producing plasmid pMS2KI. (5) A 2621-bp amplicon, from pMS2KI, was subcloned into pET32a to form pEN2K. (6) The 5'-transcriptional element, which would be translated into the Flag tag, was deleted from pEN2K using the SLIM method [40]. (7) By using SLIM method, an element encoding a stretch of six histidines was inserted into *caroS2I *to form pEH2KI. (8) A 484-bp amplicon was subcloned into pGEM T-easy vector to form pGS2I. (9) A273-bp fragment of the *caroS2I *gene was amplified from pGS2I and subcloned into pET30b to form pECS2I. (10) The 3'-transcriptional element, which would be translated to (His)_6_-Flag, was deleted from pES2I using the SLIM method. **Figure S6. Alignment of the deduced amino acid sequences of carocin S2 with those of homologous domains of bacteriocins**. The potential TonB-binding motif is shown by red underline. (A) The N-terminal translocation domain of CaroS2K (Met1 to Pro314) has homology to carocin D and colicin E3. (B) The killing domain of CaroS2K (Asp677 to carboxyl terminus) has homology to the minimal tRNase domain of colicin D and klebicin D. (C) The deduced amino acid of immunity protein of CaroS2I has homology to colicin D and klebicin D. **Figure S7. The gene and deduced amino acid sequence of *carocin S2 *shows in the study**. The sequence was truncated form pMS2KI. The underline shows the putative promoter. **Figure S8. Schematic representation of thermal asymmetric interlaced PCR (TAIL-PCR) **were manipulated according to the method of Liu and Whittier, but the annealing temperature was decreased from 63℃ to 60℃ for specific primers [37,23]. Amplifying the unknown DNA fragment are the specific primers which are complementary to the known sequence (Tn5) and the arbitrary degenerate primers which could be complementary to the opposite unknown site. The specific primers (SP) are PR1, PR2, PR3, PF1, PF2, PF3, and TF1-2S1 to TF1-2A6 primers for opposite direction (Additional file 1, Table S1). In addition, the arbitrary degenerate primers (AD) N1, N2, and N3 were respectively used as simultaneous PCR amplification (see above). Click here for file
