# Supplementary material for: Cloning, purification, and functional characterization of Carocin S2, a ribonuclease bacteriocin produced by Pectobacterium carotovorum
Source: BMC Microbiol. 2011 May 12;11:99. doi: 10.1186/1471-2180-11-99 (PMC3120645; doi:10.1186/1471-2180-11-99)
Supplement: Additional file 1 — Figure S1. Analysis of Tn5 insertional mutants by southern blotting. Lane M, the HindIII-digested λ DNA marker; the genomic DNA of strains were loading as follows: lane 1, TF1-2; lane 2, F-rif-18; lane 3, 3F3; lane 4, TF1-1. Lane 5, the construct pGnptII that contain the detect probe DNA nptII. The result shows that TF1-2 and TF1-1 was a Tn5 insertional mutant. Figure S2. The construct pMS2KI was cloned from genomic DNA library and screening by southern blotting with TF1-2 probe. By southern blotting, it showed that the carocin S2 has been cloned to form pMS2KI. Figure S3. The total RNA of SP33 were digested with Carocin S2 and electrophoresis as follows: lane 1, RNA (1 μg); lane 2, RNA and CaroS2K (20 μg); lane 3, RNA and CaroS2I (4 μg); lanes 4 to 6 are RNA (1 μg) and CaroS2K (20 μg) with gradient concentration of CaroS2I, which were added with 4 μg (lane 4); 20 μg (lane 5); 100 μg (lane 6). All reactions were performed at 28℃ for 3 hours. Figure S4. Metal effect of In vitro hydrolysis of DNA by Carocin S2. Lane M, the HindIII-digested λ DNA marker; lane 1, the genomic DNA of SP33 only; lane 2, the EcoRI-digested genomic DNA; the genomic DNA was incubated with Carocin S2 (lane 3 to 5), or not. Magnesium acetate, nickel acetate and zinc acetate was added in buffer A (pH = 7), respectively. The reactions were performed at performed at 28℃ for 1 hour. Figure S5. Schematic representation of the cloning strategy used in this study. (1) A 543-bp amplicon was cloned into the vector pTF1 to form the pTF1-2-probe. (2) The TF1-2 probe was prepared. (3) The multi-enzyme-digested DNA fragments were obtained from F-rif-18 genomic DNA, and they were detected on southern blots. (4) Positive cDNA was cloned into the carocin-producing plasmid pMS2KI. (5) A 2621-bp amplicon, from pMS2KI, was subcloned into pET32a to form pEN2K. (6) The 5'-transcriptional element, which would be translated into the Flag tag, was deleted from pEN2K using the SLIM method [40]. (7) By using SLIM method [file 1471-2180-11-99-S1.DOC]

**Additional File**

**Additional file 1**


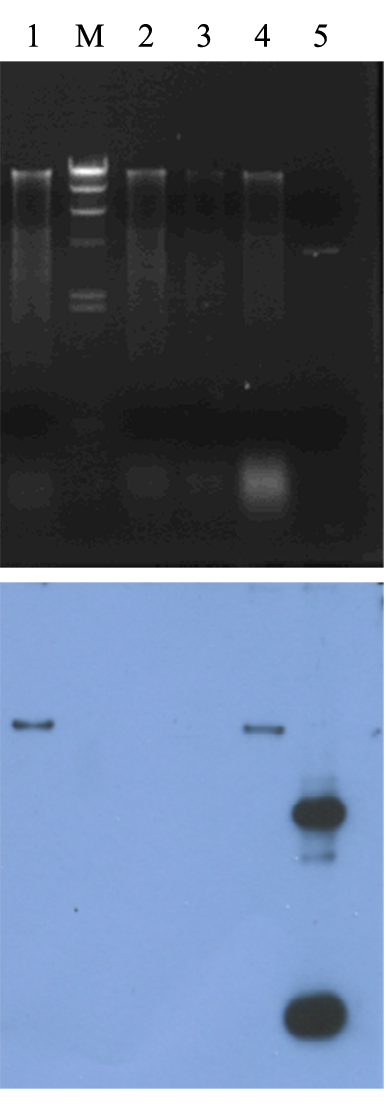


Figure S1. **Analysis of Tn5 insertional mutants by southern blotting.** Lane M, the HindIII-digested λ DNA marker; the genomic DNA of strains were loading as follows: lane 1, TF1-2; lane 2, F-rif-18; lane 3, 3F3; lane 4, TF1-1. Lane 5, the construct pGnptII that contain the detect probe DNA nptII. The result shows that TF1-2 and TF1-1 was a Tn*5* insertional mutant.


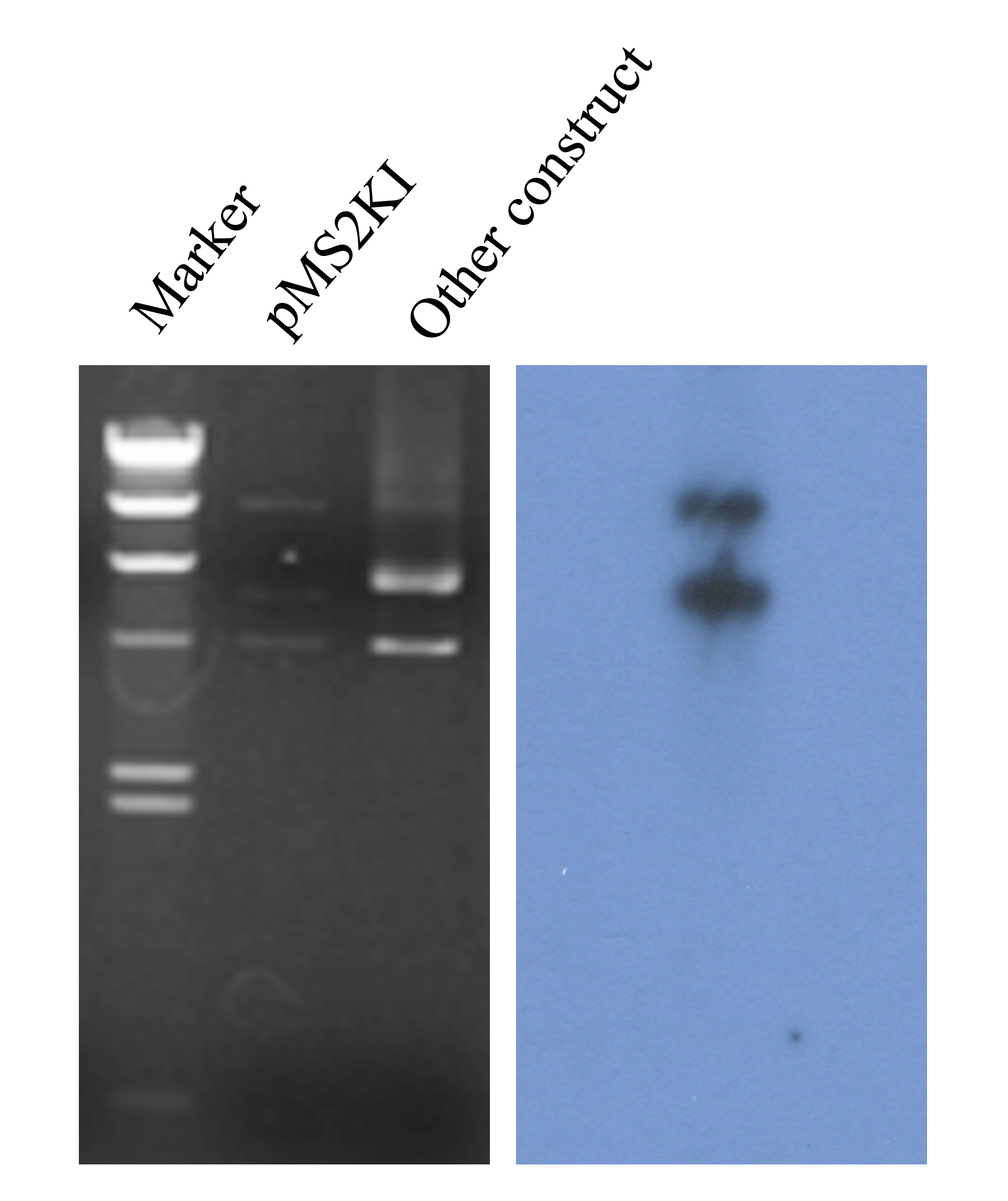


Figure S2. **The construct pMS2KI was cloned from genomic DNA library and screening by southern blotting with TF1-2 probe.** By southern blotting, it showed that the *carocin S2* has been cloned to form pMS2KI.


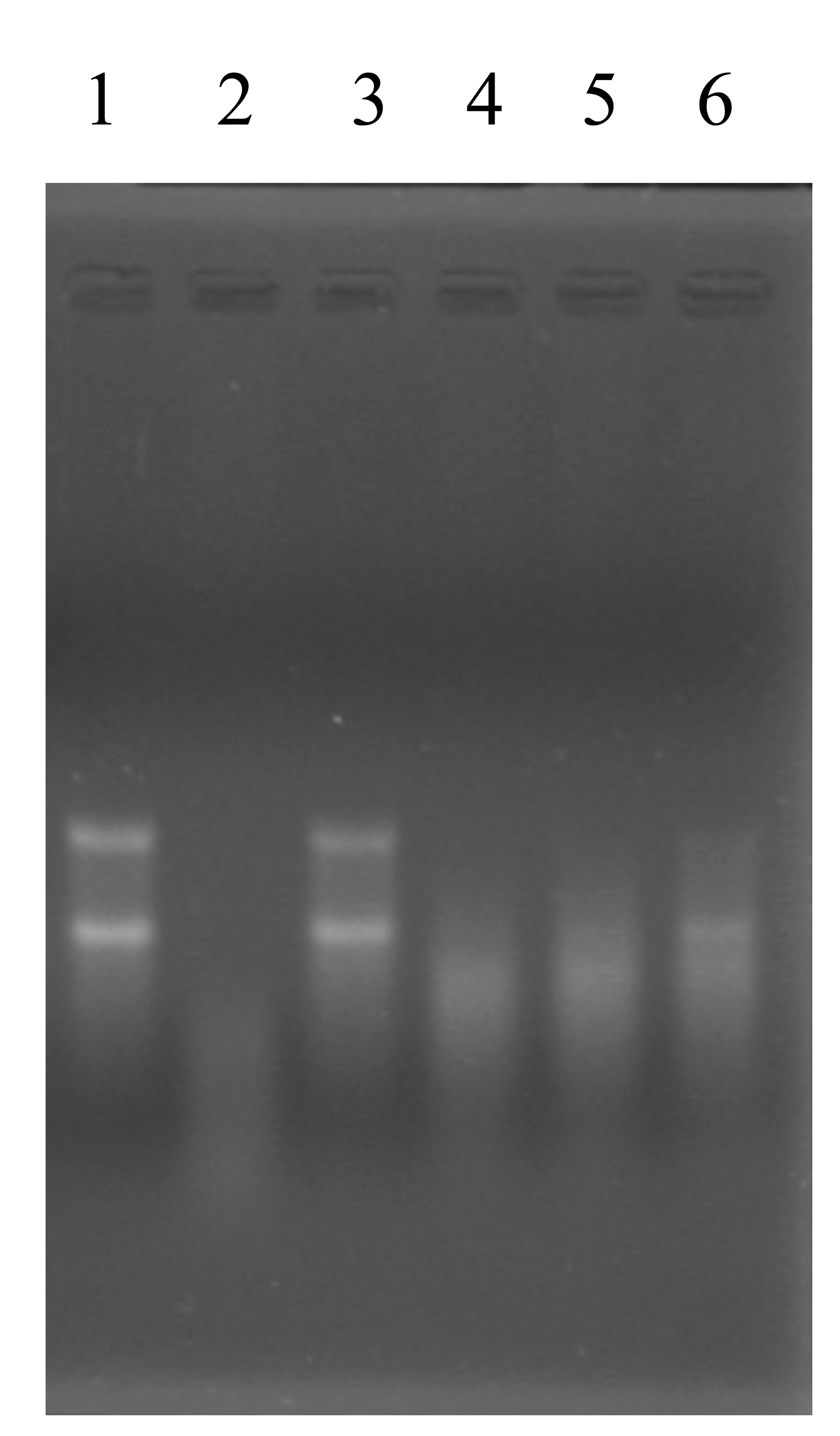


Figure S3. **The total RNA of SP33 were digested with Carocin S2 and electrophoresis** as follows: lane 1, RNA (1μg); lane 2, RNA and CaroS2K (20μg); lane 3, RNA and CaroS2I (4μg); lanes 4 to 6 are RNA (1μg) and CaroS2K (20μg) with gradient concentration of CaroS2I, which were added with 4μg (lane 4); 20μg (lane 5); 100μg (lane 6). All reactions were performed at 28℃ for 3 hours.


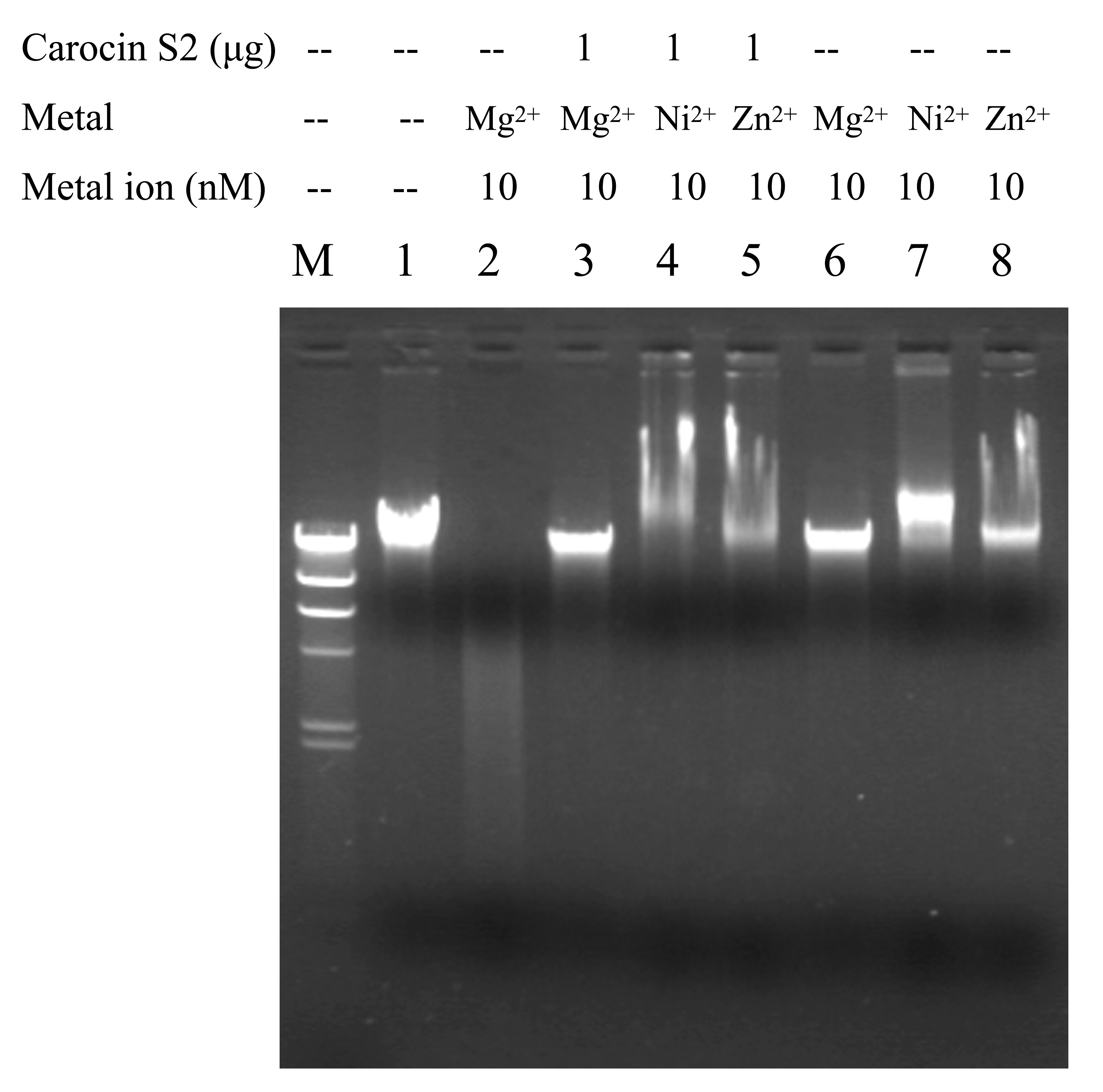


Figure S4. **Metal effect of In vitro hydrolysis of DNA by Carocin S2.** Lane M, the *Hind*III-digested λ DNA marker; lane 1, the genomic DNA of SP33 only; lane 2, the *Eco*RI-digested genomic DNA; the genomic DNA was incubated with Carocin S2 (lane 3 to 5), or not. Magnesium acetate, nickel acetate and zinc acetate was added in buffer A (pH = 7), respectively. The reactions were performed at performed at 28℃ for 1 hour.


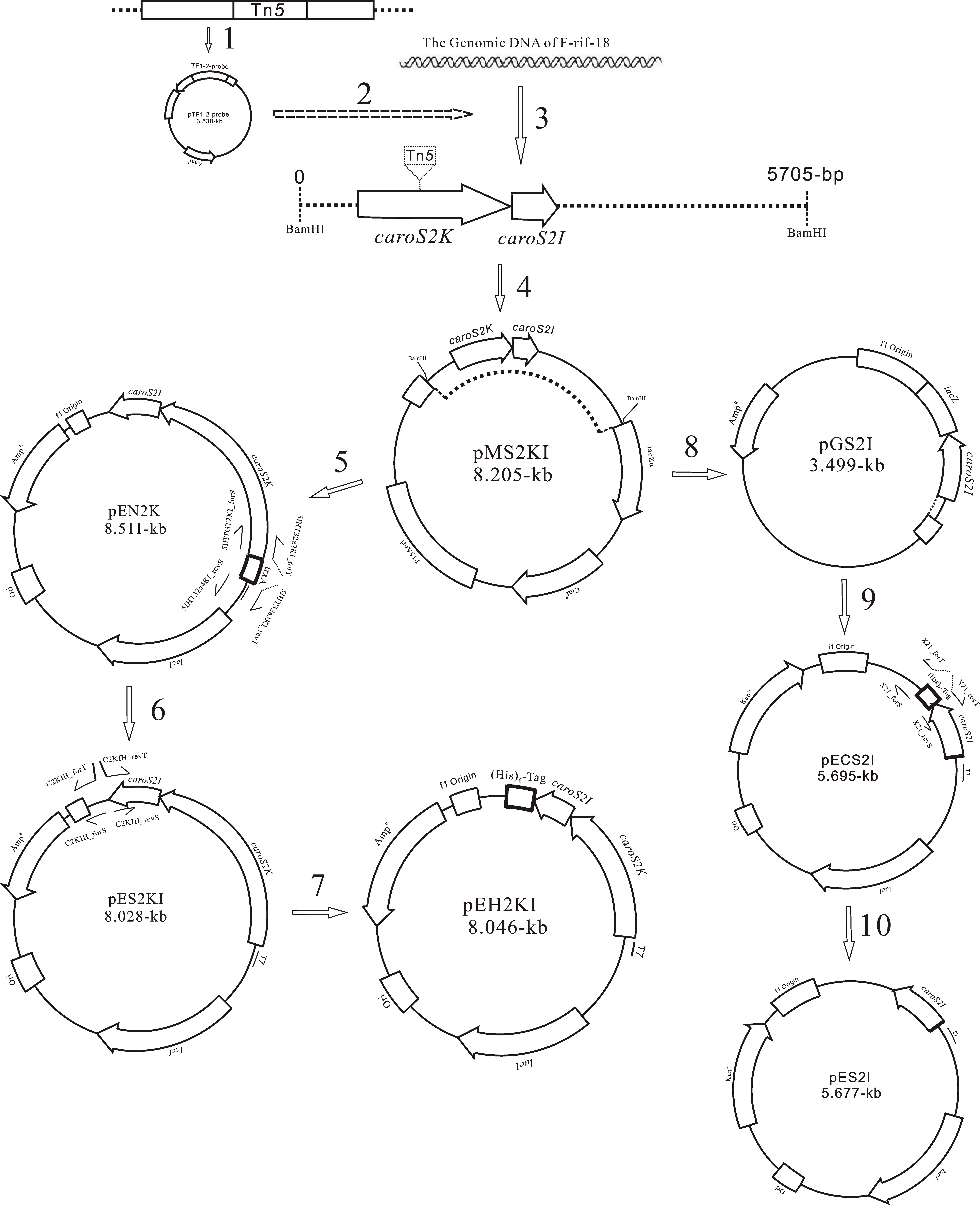


Figure S5. **Schematic representation of the cloning strategy used in this study**. (1) A 543-bp amplicon was cloned into the vector pTF1 to form the pTF1-2-probe. (2) The TF1-2 probe was prepared. (3) The multi-enzyme-digested DNA fragments were obtained from F-rif-18 genomic DNA, and they were detected on southern blots. (4) Positive cDNA was cloned into the carocin-producing plasmid pMS2KI. (5) A 2621-bp amplicon, from pMS2KI, was subcloned into pET32a to form pEN2K. (6) The 5’-transcriptional element, which would be translated into the Flag tag, was deleted from pEN2K using the SLIM method. (7) By using SLIM method, an element encoding a stretch of six histidines was inserted into *caroS2I* to form pEH2KI. (8) A 484-bp amplicon was subcloned into pGEM T-easy vector to form pGS2I. (9) A273-bp fragment of the *caroS2I* gene was amplified from pGS2I and subcloned into pET30b to form pECS2I. (10) The 3’-transcriptional element, which would be translated to (His)_6_-Flag, was deleted from pES2I using the SLIM method.


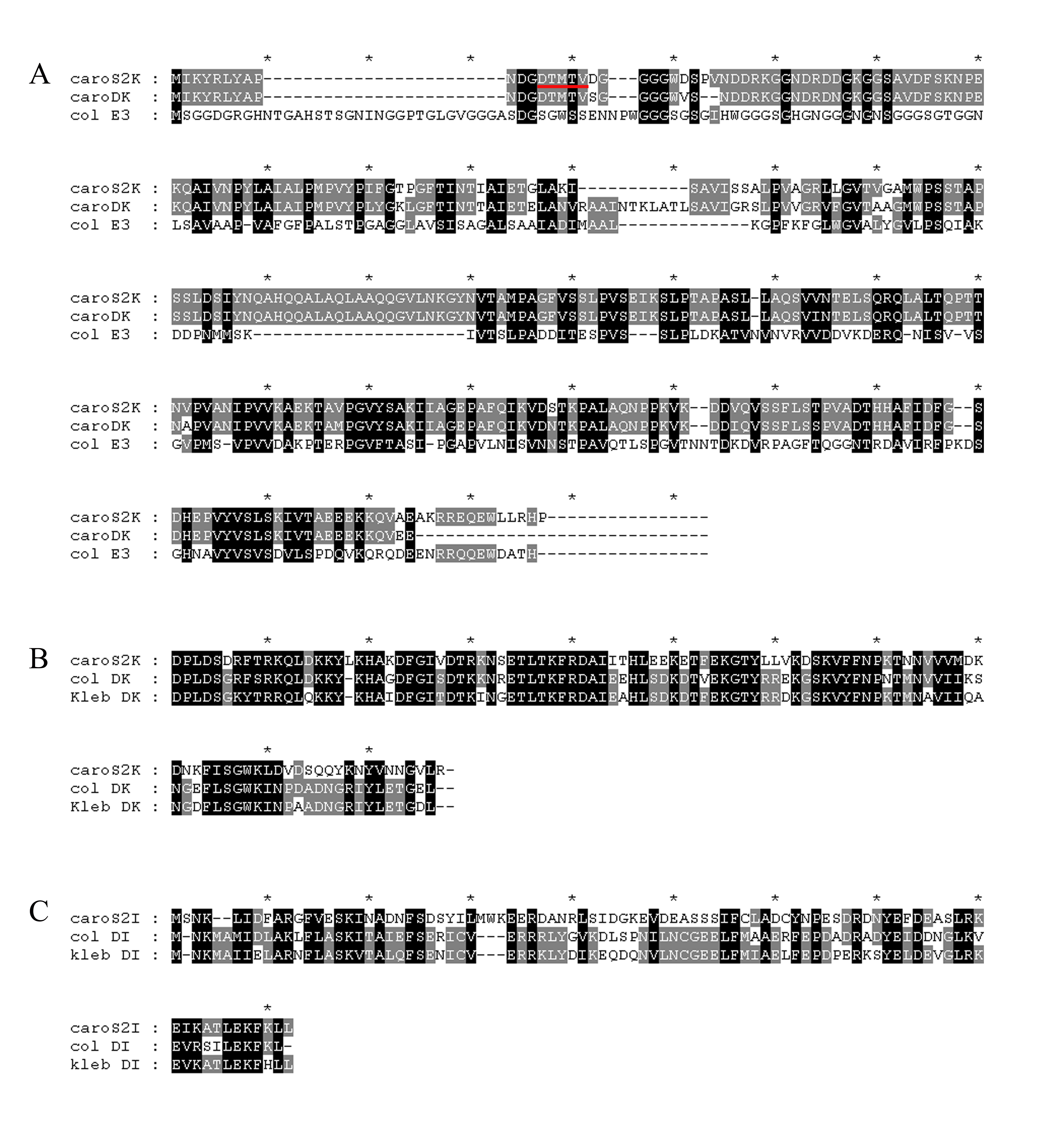


Figure S6. **Alignment of the deduced amino acid sequences of carocin S2 with those of homologous domains of bacteriocins.** The potential TonB-binding motif is shown by red underline. (A) The N-terminal translocation domain of CaroS2K (Met1 to Pro314) has homology to carocin D and colicin E3. (B) The killing domain of CaroS2K (Asp677 to carboxyl terminus) has homology to the minimal tRNase domain of colicin D and klebicin D [37,39]. (C) The deduced amino acid of immunity protein of CaroS2I has homology to colicin D and klebicin D.

GGATCCGCAGTGCGCTGGGTACGCCGAAAGCGCCGGATGAAATGACGCCAGAAGAACAACAGGCGGCTCAGCAACAGCAACAGTTGCAGGCACAGCAGCAAGACTTGGCGATGCGTGAGATTGCTGGGAAAGTGGCGAAGCTGGAAGCCGAGGCCCAGCGCATTTCCGCTGCGGCTCAGCGTGAGCAAACCTTAACGAACAGCCAGCGCTTTGATGATGCGAAAACGCAGGCGGAAACAGGTCGTATCCTTCAGGATATGGAGAATATGACGCGGGAAATTGAGGTGTTGAATGCACAGATGATGCAGGGCATTCAGGGACAAATTGATAAAATAGATTTTTAAAATCAGTTTATTAACTGAGTTTATGGAATTAGGCGAATGCTTCAGCTTGGTCAGAGAAGTCTCTATAATCTCAATACTGTATAAAAATACAGATATGGCATTTTTTGATGGAGAACTTCAATG ATT AAG TAC CGT TTA TAT GCT CCA AAT

M I K Y R L Y A P N

GAT GGA GAT ACA ATG ACC GTG GAT GGT GGC GGT GGT TGG GAC TCA

D G D T M T V D G G G G W D S

CCA GTG AAC GAT GAC CGC AAA GGT GGT AAT GAC AGG GAC GAT

P V N D D R K G G N D R D D

GGC AAA GGT GGT TCT GCC GTT GAT TTT AGT AAA AAT CCA GAA AAG

G K G G S A V D F S K N P E K

CAG GCT ATC GTT AAT CCC TAT TTG GCA ATC GCG CTA CCG ATG CCG

Q A I V N P Y L A I A L P M P

GTC TAT CCT ATT TTT GGA ACG CCA GGA TTT ACA ATA AAT ACG ATA

V Y P I F G T P G F T I N T I

GCA ATT GAG ACG GGC CTC GCC AAA ATT AGT GCA GTA ATT AGC AGC

A I E T G L A K I S A V I S S

GCG CTC CCC GTC GCT GGG CGG CTA TTG GGT GTT ACT GTA GGT GCA

A L P V A G R L L G V T V G A

ATG TGG CCT TCC AGT ACC GCT CCC AGC AGT CTC GAT TCT ATA TAC

M W P S S T A P S S L D S I Y

AAT CAG GCA CAC CAG CAG GCT CTA GCT CAG TTA GCT GCC CAG CAG

N Q A H Q Q A L A Q L A A Q Q

GGC GTG TTA AAT AAA GGG TAT AAC GTT ACA GCA ATG CCT GCG GGT

G V L N K G Y N V T A M P A G

TTC GTC AGC AGT TTG CCT GTC AGT GAA ATC AAA TCA TTG CCA ACA

F V S S L P V S E I K S L P T

GCT CCC GCC AGT TTA CTG GCA CAA AGT GTG GTT AAT ACC GAA CTT

A P A S L L A Q S V V N T E L

TCC CAG CGT CAA CTG GCT CTT ACT CAG CCC ACG ACG AAT GTA CCA

S Q R Q L A L T Q P T T N V P

GTT GCG AAT ATC CCC GTA GTT AAG GCT GAG AAA ACG GCA GTG CCT

V A N I P V V K A E K T A V P

GGC GTG TAT TCA GCG AAA ATT ATT GCT GGT GAA CCT GCA TTC CAA

G V Y S A K I I A G E P A F Q

ATC AAG GTC GAT AGT ACC AAA CCT GCT TTG GCG CAG AAT CCT CCG

I K V D S T K P A L A Q N P P

AAA GTA AAA GAT GAT GTC CAA GTC TCT TCT TTC CTT TCC ACG CCG

K V K D D V Q V S S F L S T P

GTG GCT GAT ACG CAC CAT GCA TTT ATT GAT TTT GGC AGC GAC CAT

V A D T H H A F I D F G S D H

GAA CCT GTA TAC GTG TCT CTT TCG AAG ATC GTG ACA GCC GAG GAA

E P V Y V S L S K I V T A E E

GAG AAA AAA CAG GTT GCA GAG GCC AAA CGC CGT GAG CAG GAG

E K K Q V A E A K R R E Q E

TGG TTG TTG AGA CAT CCA ATT ACC GCT GCG GAG CGA AAA TTA ACT

W L L R H P I T A A E R K L T

GAA ATC CAC CAA GTG ATC TCT TTT GCT CAA CAG CTA AAA GAA AGC

E I H Q V I S F A Q Q L K E S

TCT GCC GCA ACC ATT TCA GGA AAA ACT AAA ACT GTT GCG GTT TAC

S A A T I S G K T K T V A V Y

CAA GAA CAG GTG AAT ACC GCT GCG AAA AAT CGC GAC AAT TTT TAT

Q E Q V N T A A K N R D N F Y

AAT CAA AAT AGA GGT CTG TTA AGT GCG GGT ATA ACT GGG GGA CCG

N Q N R G L L S A G I T G G P

GGA TAT CCT ATT TAT CTC GCT TTA TGG CAA ACG ATG AAT AAT TTT

G Y P I Y L A L W Q T M N N F

CAT CAG GCT TAT TTC AGA GCA AAT AAT GCA TTG GAA CAA GAG AGT

H Q A Y F R A N N A L E Q E S

CAT GTT CTG AAC CAG GCT CGT TCT GAT CTC GCT AAG GCC GAG CAA

H V L N Q A R S D L A K A E Q

TTG CTT GCA GAG AAT AAT CTG CTT CAG GTT GAA ACG GAG CGA ACG

L L A E N N L L Q V E T E R T

CTT GCC GAA GAA AAA GAG ATA AAA CGC AAC AGG GTT AAT GTA

L A E E K E I K R N R V N V

TCA ACA TTT GGT ACA GTG CAA ACT CAA CTT AGT ACA CTG TTG TCA

S T F G T V Q T Q L S T L L S

GCA TTT TAT GCT GCT ACA TTT GGA TCT ACT ACA TCT ATT TCC CAA

A F Y A A T F G S T T S I S Q

AGT GTT CCT TCG GGG GCA TTA GCC TCT TTT TCA TAT AAG TCA CAA

S V P S G A L A S F S Y K S Q

GGG ATG ATT GGC AGC GGT AAG ATT GTT GGG AAG GAT GTC GAT ATT

G M I G S G K I V G K D V D I

TTA TTT TCC ATC CCA GTA AAA GAC ATT CCG GGA TAT AAA TCT CCT

L F S I P V K D I P G Y K S P

ACT AAC TTG GAC GAT TTA GCC AAG AAA AAT GGA AGT CTG GAT CTT

T N L D D L A K K N G S L D L

CCC ATT CGT CTG GCA TTT TCT GAT GAG AAT GGA GAA AGG GTT CTT

P I R L A F S D E N G E R V L

CGG GCA TTC AAA GCG GGT AGT CTG CGA GTC CCT TCG AGT GTC AGA

R A F K A G S L R V P S S V R

GGT GTA GCT GGT AGC TAT GAC AAA AAT ACG GGT ATT TTT AGT GCA

G V A G S Y D K N T G I F S A

AAA ATT GAC GGT GTT TCA TCT CGC CTT GTA CTG GAA AAC CCT GCG

K I D G V S S R L V L E N P A

TTT CCT CCG ACC GGA AAT GTC GGC AAT ACG GGT AAT ACT GCT CCT

F P P T G N V G N T G N T A P

GAC TAT AAA GCA TTA CTG AAT ACT GGT ATT GAT GTT AAA CCT GTT

D Y K A L L N T G I D V K P V

GAT AAA ATC ACA GTT ACG GTA ACA CCA GTT GCA GAG CCG ATG

D K I T V T V T P V A E P M

GAG TTC GAT GAT TAT ATC ATC TGG ACA CCG ACA GCA GAT GGC AGC

E F D D Y I I W T P T A D G S

GGA GTT GAA CCG ATT TAT GTG GTG TTT AAC GAT CCC TTG GAT TCA

G V E P I Y V V F N D P L D S

GAT CGG TTT ACT CGT AAG CAA CTG GAC AAA AAG TAT CTT AAA CAT

D R F T R K Q L D K K Y L K H

GCC AAA GAT TTT GGT ATT GTT GAT ACC AGA AAA AAT AGT GAA ACA

A K D F G I V D T R K N S E T

CTG ACT AAA TTT AGA GAC GCA ATT ATT ACA CAC TTA GAG GAA AAA

L T K F R D A I I T H L E E K

GAA ACT TTT GAA AAA GGG ACA TAT CTA CTT GTG AAG GAT TCA AAG

E T F E K G T Y L L V K D S K

GTT TTC TTT AAC CCG AAG ACG AAT AAT GTT GTT GTC ATG GAT AAG

V F F N P K T N N V V V M D K

GAT AAT AAA TTT ATT TCC GGT TGG AAG TTG GAT GTT GAT TCT CAG

D N K F I S G W K L D V D S Q

CAG TAT AAA AAC TAC GTT AAT AAT GGG GTG TTA AGA TGA GT AAT

Q Y K N Y V N N G V L R *

M S N

AAA CTT ATT GAT TTT GCT AGG GGT TTT GTT GAG TCT AAA ATT AAT

K L I D F A R G F V E S K I N

GCT GAT AAT TTC TCA GAT TCT TAC ATT CTC ATG TGG AAA GAA GAA

A D N F S D S Y I L M W K E E

AGG GAT GCT AAT CGG TTG TCA ATC GAT GGA AAA GAA GTA GAT

R D A N R L S I D G K E V D

GAA GCG TCG TCT AGT ATT TTT TGC TTG GCT GAT TGC TAT AAT CCA

E A S S S I F C L A D C Y N P

GAG TCG GAT CGT GAT AAT TAC GAA TTC GAC GAA GCT AGT TTA AGA

E S D R D N Y E F D E A S L R

AAA GAA ATA AAA GCC ACG TTG GAA AAA TTC AAA CTT CTT TGATAT

K E I K A T L E K F K L L *

CTCTTGCGTGTAAGAAAGATACGCGCTAAATTCGCGAAAGATACCGGAGTATGCCTTAAAAAGCTGCTCCGGTTTTTTTATACCACAAAATCAGTATCGGCCACCTTCGGGTGGCTTTTTTGTTTGTGCCGATAAGCGTTTTTGTTAGAGCGCTTATTCGCATGGGCAGCGATACGCCTTTTCCCTT

Figure S7. **The gene and deduced amino acid sequence of *carocin S2* shows in the study.** The sequence was truncated form pMS2KI. The underline shows the putative promoter.


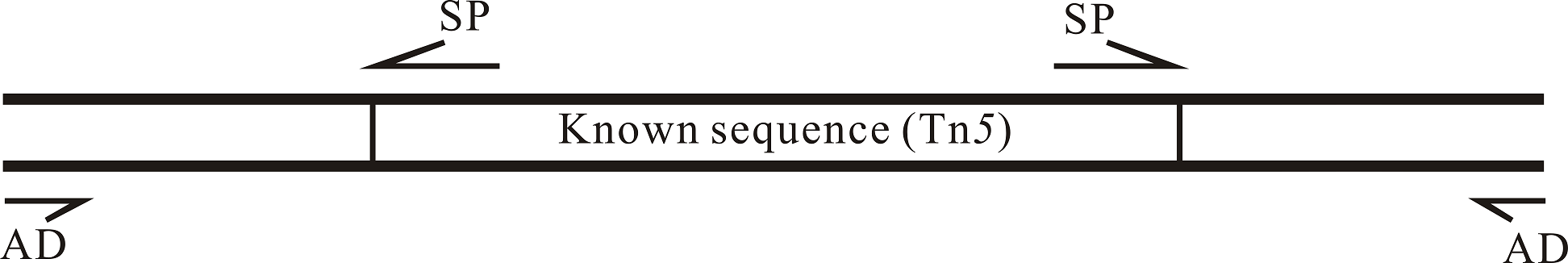


Figure S8. **Schematic representation of thermal asymmetric interlaced PCR** (TAIL-PCR) were manipulated according to the method of Liu and Whittier [27], but the annealing temperature was decreased from 63℃ to 60℃ for specific primers [22]. Amplifying the unknown DNA fragment are the specific primers which are complementary to the known sequence (Tn5) and the arbitrary degenerate primers which could be complementary to the opposite unknown site. The specific primers (SP) are PR1, PR2, PR3, PF1, PF2, PF3 [22], and TF1-2S1 to TF1-2A6 primers for opposite direction (Additional file 1, Table S1). In addition, the arbitrary degenerate primers (AD) N1, N2, and N3 were respectively used as simultaneous PCR amplification (see above).

| **Table S1.** Primers used in this study | |
| --- | --- |
| Name | Sequence |
| TF1-2S1 | CCATTCGTCTGGCATTTTGTC |
| TF1-2S2 | TTCTTCGGGCATTCAAAGCG |
| TF1-2S3 | CTTCGAGTGTCAGAGGTGTA |
| TF1-2S4 | CGGTGTTTCATCTCGCCTTG |
| TF1-2S5 | GGAAATGTCGGCATTACGG |
| TF1-2S6 | CGGAGTTGAACCGATTTATGTGG |
| TF1-2S7 | ATGATTGGCAGCGGTAAG |
| TF1-2S8 | TTTGCTTGGCTGATTGC |
| TF1-2S9 | CGATTCAGCCGTTTCCG |
| TF1-2P | ACGCCGTGAGCAGGAGTGGT |
| TF1-2A1 | AGGAACACTTTGGGAAATAGATG |
| TF1-2A2 | AGTAGATCCAAATGTAGCAGCA |
| TF1-2A 3 | ACTAAGTTGAGTTTGCACTGTACCA |
| TF1-2A4 | CTCTTCCTCGGCTGTCACG |
| TF1-2A5 | ATGTCTCAACAACCACTCCTGC |
| TF1-2A6 | CCACTCCTGCTCACGGCG |
| CarocinS2K_for2 | CGGTCAGGATCCATGATTAAGTAC |
| CarocinS2I_for2 | CCGGGAGGATCCATGAGTAATAAA |
| CarocinS2I_rev2 | GCGCCAAAGCTTCAAGAGATATCA |
| F-16sRNA-sense-ECC | CTGGACAAAGACTGACGCTC |
| R-16sRNA-anti-ECC | TCGCTGGCAACAAAGGATAAG |
| 5IHTGT2KI_forS | ATGATTAAGTACCGTTTATA |
| 5IHT32a3KI_revT | ATGTATATCTCCTTCTTAAAGTTAAACAAAATTATTTC |
| 5IHT32a2KI_forT | GAAGGAGATATACATATGATTAAGTACCGTTTATA |
| 5IHT32a4KI_revS | TTAAAGTTAAACAAAATTATTTC |
| X2I_forT | GGAAAAATTCAAACTTCTTTGAGATCCGGCTGCT |
| X2I_forS | TGAGATCCGGCTGCT |
| X2I_revT | AAGAAGTTTGAATTTTTCCAACGTGGCTTTTATTTC |
| X2I_revS | AACGTGGCTTTTATTTC |
